# A novel TREX1 inhibitor, VB-85680, upregulates cellular interferon responses

**DOI:** 10.1371/journal.pone.0305962

**Published:** 2024-08-23

**Authors:** Stephen Flowers, Brenda A. Petronella, Michael S. McQueney, Barbara Fanelli, Warren Eisenberg, Albert Uveges, Allison L. Roden, Scott Salowe, Venu Bommireddy, Jeffrey J. Letourneau, Chia-Yu Huang, James R. Beasley

**Affiliations:** 1 Oncoveda, A Division of Genesis Research & Development Institute, LLC, Hamilton, New Jersey, United States of America; 2 Venenum Biodesign, LLC, and Genesis Drug Discovery & Development, LLC, Hamilton, New Jersey, United States of America; National Taiwan University, TAIWAN

## Abstract

Activation of the cGAS-STING pathway plays a key role in the innate immune response to cancer through Type-1 Interferon (IFN) production and T cell priming. Accumulation of cytosolic double-stranded DNA (dsDNA) within tumor cells and dying cells is recognized by the DNA sensor cyclic GMP-AMP synthase (cGAS) to create the secondary messenger cGAMP, which in turn activates STING (STimulator of INterferon Genes), resulting in the subsequent expression of IFN-related genes. This process is regulated by Three-prime Repair EXonuclease 1 (TREX1), a 3’ → 5’ exonuclease that degrades cytosolic dsDNA, thereby dampening activation of the cGAS-STING pathway, which in turn diminishes immunostimulatory IFN secretion. Here, we characterize the activity of VB-85680, a potent small-molecule inhibitor of TREX1. We first demonstrate that VB-85680 inhibits TREX1 exonuclease activity *in vitro* in lysates from both human and mouse cell lines. We then show that treatment of intact cells with VB-85680 results in activation of downstream STING signaling, and activation of IFN-stimulated genes (ISGs). THP1-Dual™ cells cultured under low-serum conditions exhibited an enhanced ISG response when treated with VB-85680 in combination with exogenous DNA. Collectively, these findings suggest the potential of a TREX1 exonuclease inhibitor to work in combination with agents that generate cytosolic DNA to enhance the acquisition of the anti-tumor immunity widely associated with STING pathway activation.

## Background

Intracellular DNA is typically retained within the mitochondria or nucleus. Replication of endogenous retroelements, and intermediates generated from defective G1/S transition, can lead to accumulation of ssDNA in the cytoplasm, whereas accumulation of dsDNA can arise from micronuclei ruptures, mitotic failure from formation of chromatin bridges, mitochondrial dysfunction, or mitochondrial DNA instability [1] as well as from exogenous sources such as viral or bacterial infection. Regardless of source, accumulation of DNA in the cytoplasm leads to the activation of the cGAS-STING pathway and upregulation of downstream Type-I Interferon (IFN) and NF-κB signaling, which results in the priming of innate and adaptive immunity [2–6]. Although activation of an immune response is necessary to limit infection and establish anti-tumor immunity, unregulated response to self-DNA can result in a range of inflammatory autoimmune disorders. To prevent inappropriate immunostimulatory responses to self-DNA, cytosolic DNA levels are kept in check by cytosolic exonucleases.

The cytosolic exonuclease TREX1, also termed Deoxyribonuclease III (DNase III), is the most abundant member of the DEDDH family of 3’ → 5’ exonucleases. The TREX1 homodimer efficiently degrades ssDNA and dsDNA molecules that feature a 3’ overhang, thereby preventing them from activating STING-mediated IFN induction [7]. Full-length TREX1 is a 314 amino acid protein containing a C-terminal transmembrane domain that anchors it in the cytoplasmic side of the endoplasmic reticulum (ER). At the ER membrane, TREX1 inhibits cGAS activation by degrading DNA from ruptured micronuclei [8]. Untethering TREX1 from the endoplasmic reticulum interrupts the interaction between TREX1 and such micronuclei, thereby enhancing cGAS activity. TREX1-mediated degradation of cytosolic DNA and its impact on cGAS activity is also regulated by physically restricting access of TREX1 to DNA by the formation of cGAS-DNA condensates that protect the DNA [9].

Consistent with the critical role of TREX1 in preventing aberrant immune responses, mutations in TREX1 can cause functional loss or mislocalization, and are linked to a range of chronic inflammatory and autoimmune conditions in humans including: systemic lupus erythematosus (SLE), familial chilblain lupus (FCL), Aicardi-Goutières syndrome (AGS), and retinal vasculopathy with cerebral leukoencephalopathy (RVCL) [10–16]. Elevated expression of Type-I interferons and interferon-stimulated ISGs is common within most TREX1-associated autoimmune conditions. However, in cases of RVCL, there is no evidence of IFN upregulation [17]. Similarly, TREX1-deficent mice exhibit inflammatory phenotypes and reduced postnatal survival where development of inflammatory myocarditis, leads to progressive cardiomyopathy and circulatory failure by 20 weeks of age. In corresponding human disease, however inflammatory myocarditis is not common [18].

Although TREX1 functional loss in normal cells is associated with negative outcomes, increased TREX1 expression in tumor cells with inherent genetic instability may promote disease progression by limiting the acquisition of anti-tumor immunity [19]. For example, increased TREX1 expression in cervical tumor cells is associated with cervical cancer onset and/or progression [20]. Elevated TREX1 expression has also been observed in response to chemotherapeutics and ionizing radiation in various cancer cell types [21–24] resulting in decreased levels of cytosolic dsDNA, diminished activation of the cGAS-STING pathway, and consequent reduction of Type-I IFN secretion.

Based on these reported findings, pharmacologic inhibition of TREX1 activity in tumor cells is expected to generate an increase in Type-I IFN secretion (and consequently, ISG stimulation) similar to that observed with STING agonists. Indeed, TREX1-deficiency in mouse tumor models has been demonstrated to elicit a strong anti-tumor immune response [25–27] lending further support to the potential efficacy ofTREX1 inhibitors in the treatment of malignant disease. Here we demonstrate that a novel TREX1 inhibitor, VB-85680, is active against both human and murine TREX1 and that introduction of VB-85680 in combination with exogenous dsDNA increases the expression of several key ISGs involved in the innate immune response. TREX1 inhibitors such as VB-85680 may have utility in combinatorial immune-oncology treatment strategies with radiation therapy or targeted chemotherapeutics, to maximize immunogenicity arising from increased levels of cytosolic DNA.

## Material and methods

### Compound synthesis

TREX1 inhibitors VB-85680, VB-86087 and VB-85662 were synthesized as described previously [28, 29].

### Preparation of HEK293T overexpression lysates

TREX1 overexpression in HEK293T cells (ATCC CRL-3216) was achieved via transient transfection of pcDNA3.1 plasmids harboring either wild-type full-length mouse TREX1 (GenScript OMu22134C) or the catalytically inactive TREX1 D18N mutant (GenScript OMu22134M). Each insert was cloned into pcDNA3.1(+) by HindIII/BamHI. Transfections were carried out using Lipofectamine™ LTX with PLUS™ Reagent in Opti-MEM^®^ (Thermo Fisher) in a 6-well dish according to the protocol recommended by the manufacturer. An empty-vector transfection was performed in parallel for use as a negative control. Transfected cells were harvested 48 hours after transfection and washed with PBS before being pelleted and stored at -80°C prior to use. Cell pellets were thawed on ice and resuspended in PBS. Lysates were prepared by sonication in an ultrasonic water bath (6 cycles of 2-minute sonication alternated with icing). Total protein concentration in each lysate was determined using the Pierce™ BCA Protein Assay kit (Thermo Fisher) with BSA as the standard.

### Measurement of exonuclease activity in HEK293T lysates

Exonuclease activity in HEK293T overexpression lysates was evaluated by measuring the increase in fluorescence resulting from exonuclease-catalyzed cleavage of a quencher from the 3’ end of a dual-labeled DNA oligonucleotide (5’FAM-CCA CGA GAG CGT-BHQ1-3’). To evaluate the effect of compounds on exonuclease activity, test compounds were serially-diluted (11-point, 3-fold) from 10 mM stock solutions and delivered to 384-well low-volume assay plates in 80 nL DMSO using an acoustic dispenser. Next, 4 μL of mouse wild-type TREX1 (10 ng/μL), and TREX1 D18N or empty-vector (200 ng/μL) HEK293T lysates diluted in assay buffer (20 mM Tris pH 7.5, 5 mM MgCl_2_, 100 μg/mL BSA, 0.002% Triton X-100, 2 mM DTT), were added to the assay plate. After incubating for 30 minutes, 4 μL of dual-labeled DNA oligonucleotide (500 nM) in assay buffer was added to initiate the exonuclease reaction. The reaction was allowed to proceed for 45 minutes at room temperature prior to the addition of 4 μL of 150 mM EDTA to halt exonuclease activity. Assay plates were equilibrated for an additional 30 minutes prior to reading on an EnVision Plate Reader (Perkin Elmer) to measure fluorescence emission at 535 nm following excitation at 485 nm. Fluorescence was plotted as a function of log molar compound concentration and fit to a four-parameter dose-response equation to determine compound IC_50_.

### Endogenous TREX1 exonuclease activity assay

Endogenous TREX1 activity was measured using the BioVision 3’ to 5’ Exonuclease Activity Assay Kit (Abcam ab273269) according to the manufacturer’s protocol. Briefly, THP1-Dual™ (Invivogen thpd-nfis), THP1-Dual™ TREX1 KO (Invivogen thpd-nfis) or 4T1 (ATCC CRL-2539) cells were suspended at a density of 1 million cells/mL in the lysis buffer provided. For each set of lysates, 6 μg of protein in 20 μL assay buffer was added per well and preincubated with 5 μL of serially-diluted VB-85680 or VB-85662 (5 nM to 100 μM) for 30 minutes at 37°C. After the preincubation period, 25 μL of the provided probe was added and the plate was incubated overnight at room temperature in a humidified chamber. The fluorescence signal was read using an EnVision plate reader (Perkin Elmer). The monochromators for excitation and emission were set to 304 nm and 369 nm, respectively. An IC_50_ for each compound was determined by plotting the signal as a function of log molar compound concentration and fitting to a four-parameter logistic equation in GraphPad Prism 5.0.

### Analysis of gene expression by RT-qPCR

THP1-Dual^TM^ cells were treated with 10 μM test compound in the presence or absence of 1,200 ng/mL VACV-70 oligonucleotide (InvivoGen), or with VACV-70 alone, for 24 hours. Cells were harvested by centrifugation and washed with phosphate buffered saline (PBS). Total RNA was isolated using the RNeasy^®^ mini kit (Qiagen) and genomic DNA was degraded using DNase 1 (Thermo Scientific). Total RNA was quantified using a Nanodrop^®^ spectrophotometer. For each sample, cDNA was synthesized from 1 μg RNA using the SuperScript^TM^ III First-Strand synthesis system (Invitrogen). Target gene expression levels were determined relative to GAPDH by qPCR using TaqMan^TM^ gene expression assays (Applied Biosystems^TM^) run on a Stratagene Mx3005P qPCR system with standard cycling parameters. Gene expression data were analyzed by the comparative C_T_ method and statistical analysis was performed using one-way ANOVA and Tukey’s Multiple Comparison Test in GraphPad Prism 5.0. Primers are listed in Table 1.

**Table 1:** List of Taqman assays used in this study.

Primer	Assay IDs
GAPDH	Hs02786624_g1
IFI27	HS01086373_g1
IFI44	Hs00197427_m1
IFI44L	Hs00915292_m1
IFI6	Hs00242571_m1
IFIT1	Hs01675197_m1
IFIT2	Hs01933738_s1
IFIT3	Hs01922752_s1
ISG20	Hs00158122_m1
OAS3	HS00196324_m1
OASL	Hs00984387_m1
TRIM22	Hs00232319_m1
USP18	Hs00276441_m1

### RNA-sequencing

Total RNA was isolated from Human THP1-Dual™ cells as described above and sent to GENEWIZ (South Plainfield, NJ 07080) for RNA-sequencing. Sequence reads were trimmed to remove possible adapter sequences and nucleotides with poor quality using Trimmomatic v.0.36 (USADEL LAB). The trimmed reads were mapped to the *Homo sapiens* GRCh38 reference genome available on ENSEMBL using the STAR aligner v.2.5.2b (https://github.com/alexdobin/STAR). BAM files were generated. Unique gene hit counts were calculated by using “feature counts” from the Subread package v.1.5.2 (https://subread.sourceforge.net/). Hit counts were summarized and reported using the “gene id” feature of the annotation file. Only unique reads that fell within exonic regions were counted. Since a strand-specific library preparation was performed, the reads were strand-specifically counted. After extraction of gene hit counts, the gene hit counts table was used for downstream differential expression analysis. Using DESeq2 (Bioconductor), a comparison of gene expression between defined groups of samples was performed. The Wald test was used to generate p-values and log2 fold-changes. Genes with an adjusted p-value < 0.05 and absolute log2 fold-change > 1 were called as “differentially-expressed” genes for each comparison. For Gene ontology “GO” analysis, differentially expressed genes were clustered by their gene ontology and the enrichment of gene ontology terms was tested using Fisher’s exact test (GeneSCF v1.1-p2).

### Immunoblotting

THP1-Dual™ cells (750,000) were washed and harvested in PBS [pH 7.0] and lysed in RIPA lysis buffer containing protease and phosphatase inhibitors (Roche). Proteins (10 μg lysate per lane) were separated by PAGE (4 to 18% and 4 to 20%; Bio-Rad), transferred to iBlot^TM^ nitrocellulose membrane (Invitrogen), and visualized using HRP detection. Antibodies used in this study included IFIT1 (Cell Signaling #14769), STING (Cell Signaling # 13647), mouse cGAS (Cell Signaling #31659), human cGAS (Cell Signaling #15102) mouse TREX1 (Santa Cruz #sc133112), human TREX1 (Santa Cruz #sc-271870), β-Actin (Abcam #ab20272), and α-Tubulin (Abcam #ab40742).

### THP1-Dual™ reporter assay

Three days prior to the assay, THP1-Dual™ cells were transferred to a T75 flask at a density of 3.0 x 10^5^ cells/mL in 20 mL of reduced-serum medium (RPMI 1640 containing 1% Heat-Inactivated FBS, 1x Glutagro, 10 mM HEPES, 1 mM sodium pyruvate, 1x Pen/Strep, 100 μg/mL Normocin, 100 μg/mL Zeocin, 10μg/mL Blasticidin). On the day of assay, cells were collected by centrifugation, washed in Dulbecco’s Phosphate Buffered Saline, and resuspended in reduced-serum medium at 3.3 x 10^5^ cells/mL. G3-YSD DNA oligonucleotide (InvivoGen) at 1 mg/mL was diluted 100-fold into the LyoVec^TM^ (InvivoGen) transfection reagent and incubated at ambient temperature for 50 min to allow complex formation. Test compounds were serially-diluted using a 3-fold dilution series (final concentration ranging from 0.5 nM to 30 μM) in DMSO. Compounds were transferred to a standard 96-well cell culture plate via acoustic dispensing of 0.54 μL per well. The final DMSO concentration was 0.3%. Serum-starved cells were batch-transfected with the G3-YSD/LyoVec complex at a final DNA concentration of 10 ng/mL for 60 minutes at ambient temperature before dispensing 180 μL (60,000 cells) per well into the assay plate containing compounds. The assay plate was then incubated in a humidified chamber at 37°C and 5% CO_2_ for three days. An aliquot of 10 μL was removed from each well and combined with 10 μL of the QUANTI-Luc™ Gold (InvivoGen) luciferase detection reagent, in a white 384-well plate. Luminescence was read using an EnVision plate reader (Perkin Elmer). An IC_50_ for each compound was determined by plotting the signal as a function of log molar concentration and fitting to a four-parameter logistic equation in GraphPad Prism 5.0.

For G150 experiments, cells were plated in 96-well plates as described above. Cells were simultaneously treated with 1μM of the TREX1 inhibitor VB-86087, 10 ng/mL G3-YSD/LyoVec, and a 3-fold dilution series of G150 (Selleckchem #S8944) for 72 hours.

### WST-1 cellular proliferation assay

THP1-Dual™ cells were plated at 60,000 cells per well in 150 μL medium (RPMI 1640 containing either 10% or 1% Heat-Inactivated FBS, 1x Glutagro, 10 mM HEPES, 1 mM sodium pyruvate, 1x Pen/Strep, 100 μg/mL Normocin, 100 μg/mL Zeocin, 10μg/mL Blasticidin) and allowed to grow for 48 hours. Cells were then transfected with 1.2 μg/mL VACV-70 in 50 μL and incubated for an additional 18 hours. Following incubation, 10 μL of WST-1 (Abcam #ab155902) was added to each well, and 1.5 hours later absorbance at 450 nm was read using an EnVision plate reader (Perkin Elmer)

## Results

### VB-85680 inhibits TREX1 activity in both mouse and human cell lysates

Prior studies demonstrated that VB-85680 inhibits the exonuclease activity of purified truncated human and mouse TREX1 constructs, with similar potency [28, 29], but neither its ability to inhibit full-length TREX1, nor its activity against endogenous TREX1 exonuclease has yet been demonstrated. To confirm the activity of VB-85680 against cellular TREX1 enzymes independent of any barriers to cellular permeability, we tested VB-85680 for inhibition of exonuclease activity in cell lysates, using two different assay systems.

**Fig 1:**
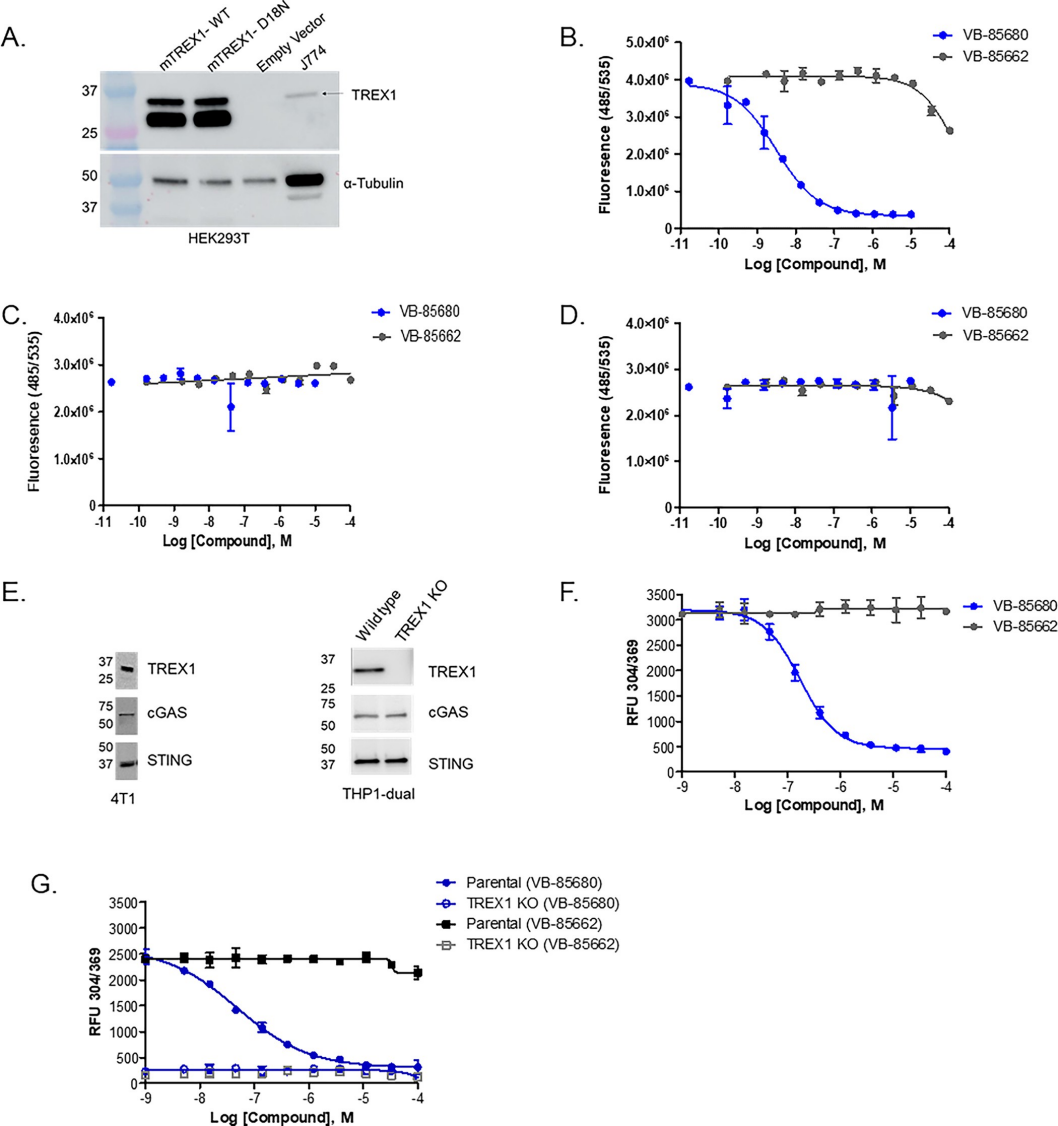
VB-85680 inhibits full-length mouse and human TREX1 in cellular lysates. **A)** TREX1 Western blot demonstrating overexpression of both wild-type mouse TREX1 and mouse TREX1 carrying the D18N inactivating mutation. Endogenous mouse TREX1 present in a J774 cellular lysate is shown as reference to confirm the size of the upper band as full-length mouse TREX1. α-tubulin was used as a loading control. **B–D)** Exonuclease assays performed with HEK293T mouse TREX1 overexpression or empty vector lysates demonstrate the effects of VB-85680 and VB-85662 on **B)** wild-type mouse TREX1 (5 ng/μL total protein), **C)** Catalytically inactive mouse TREX1 D18N (100 ng/μL total protein) and, **D)** endogenous exonuclease activity (100 ng/μL total protein). **E)** Western blot for endogenous levels of cGAS,STING and TREX1 in both 4T1 and THP1-dual cells. **F)** Endogenous exonuclease activity in 4T1 cell lysates is inhibited by VB-85680, but not by VB-85662. Compounds were titrated in the presence of 6 μg 4T1 cytosolic lysate using the 3’ to 5’ Exonuclease Activity Assay from BioVision. **G)** Inhibition of endogenous human TREX1 exonuclease activity by VB-85680 in THP1-Dual™ and THP1-Dual™ TREX1 knockout cell lysates. The exonuclease assay was carried out as described in **1E**. Error bars for all experiments represent +/- SD.

Since full-length TREX1 protein complexes are unstable, and prior attempts to purify to homogeneity have failed, we overexpressed full-length mouse TREX1 in HEK293T cells (which lack detectable expression of human TREX1, Fig 1A, empty vector lane) and performed activity assays using HEK293T lysates prepared from cells harvested 48 hours after transfection. To assess the contribution of overexpressed mouse TREX1 to the observed assay signal and rule out any interference from endogenous exonuclease activity present in HEK293T cell lysates, we also overexpressed the catalytically-inactive mouse TREX1 D18N protein in the same cell-line [13]. Lysates prepared from an empty-vector transfection served as an additional negative control. Expression of wild-type or D18N mouse TREX1 in HEK293T lysates was confirmed by Western Blot (Fig 1A). The upper bands observed in lanes corresponding to wild-type and mutant mouse TREX1 are consistent with the 34 kDa molecular weight expected for full-length mouse TREX1 and align with a band corresponding to lower levels of endogenous mouse TREX1 present in a J774 cell lysate. A lower molecular weight band was also present in the overexpression lysates and may be attributable to either proteolytic degradation or incomplete translation of full-length mouse TREX1 in HEK293T.

To assess VB-85680 inhibition against full-length mouse TREX1 in the HEK293T overexpression lysates described above, we employed the same biochemical exonuclease assay used previously to demonstrate compound inhibition against truncated TREX1 exonuclease constructs. This assay monitors the increase in fluorescence resulting from cleavage of a quencher from the 3’ end of a dual-labeled FAM-12mer-BHQ1 oligonucleotide substrate. In this assay system, inhibition of exonuclease activity manifests as a decrease in fluorescence in the presence of inhibitor. Treatment of full-length mouse TREX1 lysate with VB-85680 resulted in inhibition of exonuclease activity with an IC_50_ of 3.1 nM (Fig 1B), consistent with its reported range of potency against truncated mTREX1 (28, 29). Likewise, treatment with VB-85662, a previously reported inactive analog of VB-85680, demonstrated only modest decreases in fluorescence at the highest concentrations tested, corresponding to an IC_50_ > 100 μM against full-length TREX1. As expected, overexpression lysate corresponding to catalytically inactive mTREX1 D18N failed to show any enhancement in exonuclease activity over that observed in the “empty vector” lysate. Basal exonuclease activity in each lysate was at least 20-fold lower than that observed in wild-type mTREX1 lysates and was not inhibited by VB-85680 (Fig 1C and 1D), indicating that endogenous endonucleases did not contribute significantly to the observed assay signal, and further suggesting that VB-85680 specifically inhibits mouse TREX1 activity in this assay system.

Next, we used a 3’ to 5’ Exonuclease Activity assay (BioVision) to explore VB-85680 inhibition of endogenous mouse and human TREX1, from 4T1 lysates and THP1-Dual™ lysates, respectively. Endogenous levels of TREX1 along with STING and cGAS in both 4T1 and THP1-dual are shown in Fig 1E. VB-85680 inhibited mouse TREX1 activity in 4T1 lysates with an IC_50_ of 171.6 nM (Fig 1F) and inhibited human TREX1 activity in THP1-Dual™ lysates with an IC_50_ of 48.8 nM (Fig 1G). These IC_50_ values indicate reduced potency compared to both truncated TREX1 constructs and full-length mTREX1 in the HEK293T overexpression lysates. Such discrepancies may be related to the use of different substrates in the respective assays. As expected, VB-85662 did not inhibit either human or mouse TREX1. In lysates from a THP1-Dual™ TREX1 knockout line, no detectable exonuclease activity was observed (Fig 1G).

### VB-85680 elicits strong ISRE reporter activity in THP1-Dual™ cells conditioned with low-serum for increased TREX1 expression

Having demonstrated that VB-85680 inhibits TREX1 activity in cellular lysates, we explored conditions to assess compound activity in intact cells.

A cell-based assay was established using THP1-Dual™ cells (InvivoGen), a human monocyte cell-line that has been engineered to include stable integration of an Interferon Stimulated Response Element (ISRE)-Lucia reporter gene. Under control of an ISG54 minimal promoter containing five IFN-stimulated response elements, the Lucia gene encodes a secreted luciferase reporter protein. Activation of the cGAS-STING pathway leads to enhanced luciferase secretion in these cells.

**Fig 2:**
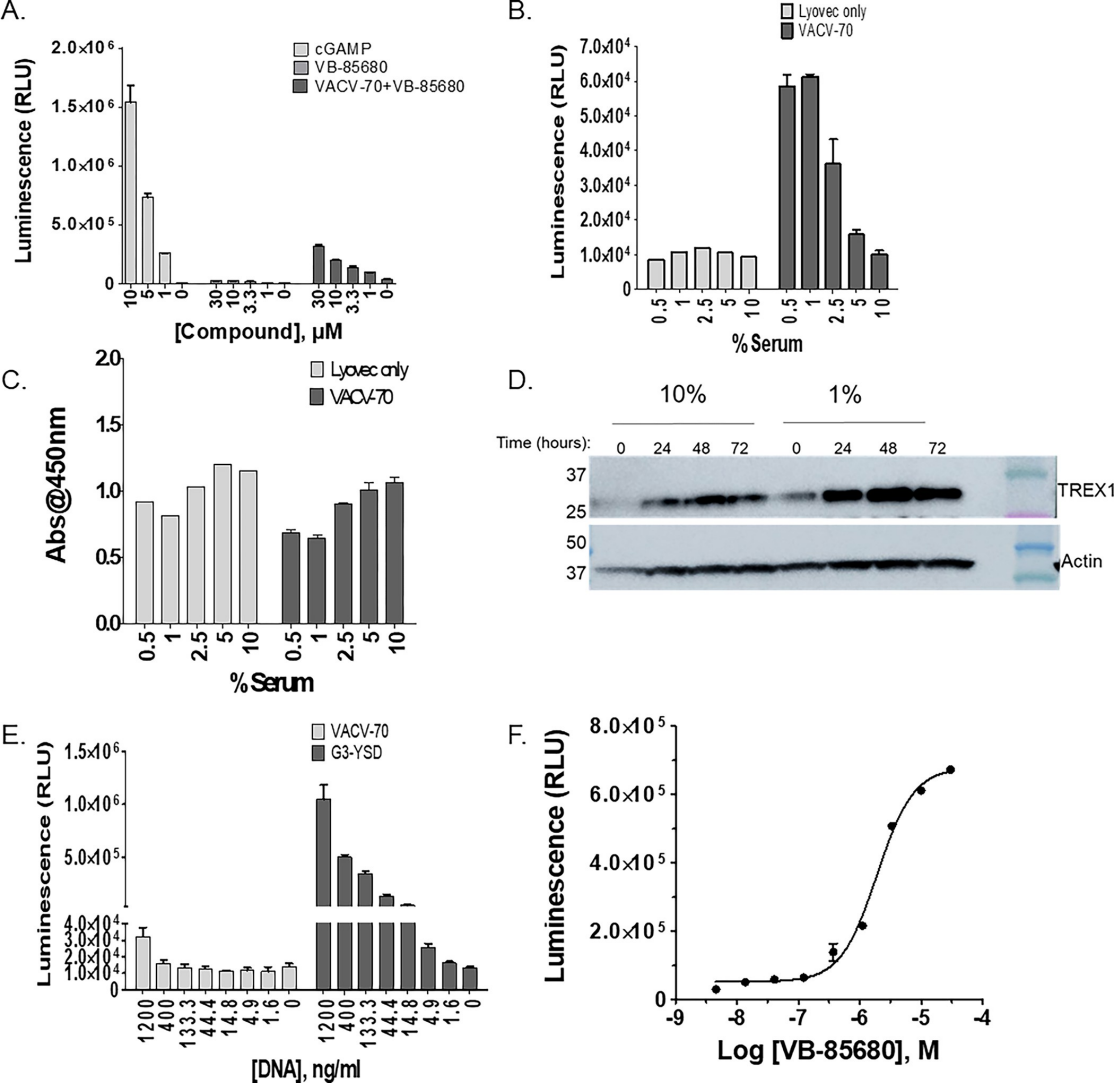
Dose response activity of TREX1 VB-85680 in THP1-Dual™ cells is enhanced under lower-serum growth conditions. **A**) Activation of the THP1-Dual™ interferon regulatory factor (IRF) pathway by VACV-70 in combination with VB-85680 was monitored by detection of Lucia luciferase by Quanti-Luc gold. Increased activity was only detected using either cGAMP alone or the combination of TREX1 inhibitor and DNA. **B)** THP1-Dual™ cells were cultured for 2-days in various serum concentrations followed by transfection with 1200 ng/mL VACV-70 for an additional 18 hours. The Quanti-Luc™ assay was used to demonstrate increased sensitivity of the reporter to exogenous VACV-70 DNA under low serum conditions. **C**) A WST1 assay was run in parallel to demonstrate the relative health of cells cultured in lower serum conditions and transfection with VACV-70 DNA. **D**) THP-1-Dual™ cells were cultured for 72 hours in either 10% serum or 1% serum. Western blotting was used to evaluate the expression of TREX1 over time in cells grown in each medium. TREX1 protein levels increased in THP1-Dual™ cells cultured under low serum conditions. **E)** The Quanti-Luc™ assay was used to assess reporter responsiveness to G3-YSD transfection relative to VACV-70. **F)** THP1-Dual™ cells were cultured for 3 days under low serum conditions. The cells were then batch-transfected with 10 ng/mL G3-YSD and treated with decreasing doses of VB-85680, starting at 30 μM. The IC_50_ of VB-85680 was determined to be 2.9 μM. Error bars for all experiments represent +/- SD.

Treatment of the THP1-Dual™ cells with VB-85680 alone did not cause any noticeable increase in secreted luciferase. Addition of VACV-70 DNA revealed a marked VB-85680 dose-dependent increase of signal, indicative of cGAS-STING pathway activation (Fig 2A). Although the combination of TREX1 inhibitor and VACV-70 resulted in an increased response, we did not observe a sigmoidal dose response pattern necessary for IC_50_ determination. cGAMP directly stimulates STING and was thus employed as a positive control.

Fang *et al*, reported that cells which were unresponsive to dsDNA in regular culture medium were responsive when cultured in lower FBS concentrations [30]. Metabolic stress (such as nutrient depletion) has also been shown to induce cytoplasmic ssDNA in human TNBC cell-lines leading to IFN responses [31]. To determine the extent to which serum conditions can sensitize the cells to TREX1 inhibition, THP1-Dual™ cells were cultured in various serum concentrations ranging from 0.5% to 10% and then transfected with VACV-70. The activation of the luciferase reporter gene in THP1-Dual™ cells was found to be ~6 fold higher under serum concentrations as low as 0.5% compared to cells grown in 10% serum (Fig 2B). Cells grown in lower serum remained viable during the assay period (Fig 2C). TREX1 expression levels can increase in response to multiple stimuli including accumulation of cytoplasmic DNA and Type-I IFN signaling. In our hands, elevated TREX1 expression was evident in THP1-Dual™ cells 24 hours following exposure to lower-serum conditions and persisted for at least 72 hours (Fig 2D). Accordingly, we chose to proceed with an assay containing 1% serum.

As a source of exogenous DNA, we recognized that VACV-70 might activate other DNA sensors in addition to cGAS and either limit activation of the cGAS-STING pathway or complicate interpretation of results. For that reason, we evaluated G3-YSD as a more specific cGAS-activating ligand that is also a known substrate of TREX1 [9, 32, 33]. Treatment with G3-YSD DNA resulted in ~32-fold higher stimulation of ISG reporter activity in THP1-Dual™ cells relative to VACV-70 DNA (Fig 2E). In THP1-Dual™ cells grown under low-serum conditions and treated with G3-YSD DNA, VB-85680 titration activated reporter signaling with a sigmoidal dose response profile corresponding to an IC_50_ of 2.9 μM (Fig 2F).

We have also confirmed that activation of reporter signaling in the presence of our TREX1 inhibitors is dependent on an intact cGAS-STING pathway. VB-86087, a potent analog of VB-85680, stimulates reporter activity with an IC_50_ of 0.25 μM in the low serum/G3-YSD THP1-Dual™ assay (S1A Fig), an effect that is potently and completely reversed when cells are co-treated with G150, an inhibitor of human cGAS activity (S1B Fig) [34].

### VB-85680 treatment elevated interferon pathway gene expression in THP1-Dual™ cells primed with VACV-70 DNA

We wished to examine global changes in RNA expression following treatment of intact cells with VB-85680, with particular focus on aspects of the IFN response. THP1-Dual™ cells were treated for 24 hours with 10 μM VB-85680, 1200 ng/mL VACV-70, or a combination of both. cGAMP (10 μM) was used as a positive control. After 24 hours of treatment, cells were collected for mRNA extraction and sequencing. Differentially-expressed genes were identified relative to untreated cells as described across all treatment combinations (Table 2).

**Table 2:** Summary of differential gene expression analysis. The results of the number of differentially expressed genes for all comparisons is provided. Each group is relative to the untreated control group.

Comparison	Upregulated	Downregulated	Total
cGAMP	410	44	454
VACV-70	0	3	3
VB-85680	27	1	28
VACV-70+ VB-85680	147	7	154

The cGAMP control cells exhibited the largest number (457) of differentially expressed genes relative to the untreated control, with 410 upregulated and 44 downregulated genes. Cells treated with both VB-85680 and VACV-70 demonstrated 147 upregulated and 7 downregulated genes when compared to untreated control cells. Cells treated with VACV-70 alone displayed only 3 differentially expressed genes, all of which were downregulated. In the VB-85680-treated cells, although 28 differentially-expressed genes were identified, including 27 which were upregulated, the log2-fold change was below the 2-fold cut off for significance. Since there were no significantly upregulated genes in either of the single-treatment groups, we focused on the comparison of cGAMP-treated cells to cells treated with the combination of VB-85680 and VACV-70.

**Fig 3:**
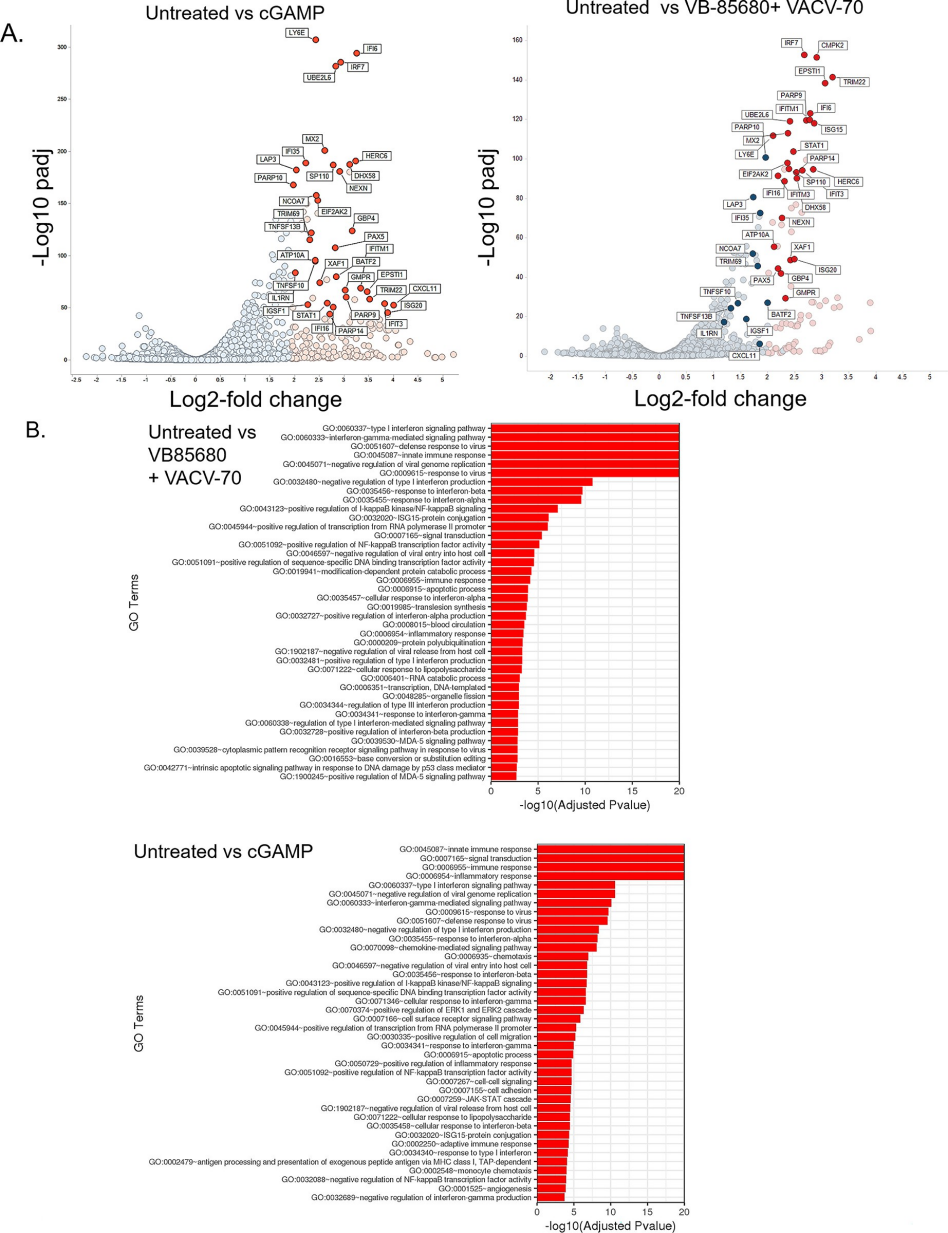
RNAseq analysis of THP1-Dual™ cells treated with the combination of VB-85680 and VACV-70 shows an increased interferon response similar to cGAMP treated cells. **A)** Global transcriptional changes for each treatment group were visualized using volcano plots. Each data point represents a single gene. The log2 fold-change of each gene is represented on the x-axis and the log10 of its adjusted p-value is on the y-axis. Genes common between the two comparisons that have an adjusted p-value less than 0.05 and a log2 fold change greater than 2 are indicated by red dots, and represent upregulated genes. Common genes with an adjusted p-value less than 0.05 and a log2 fold change less than 1 are indicated with blue dots**. B)** Gene ontology (GO) analysis of THP1-Dual™ cells treated with VB-85680 in combination with VACV-70 or cGAMP alone shows an enrichment of immune-related pathways. GO analysis shows gene ontology terms that are significantly enriched with an adjusted P-value less than 0.05 in the differentially expressed gene sets.

Volcano plots generated for cGAMP treated cells and cells treated with the combination of VB-85680 and VACV-70 illustrated upregulation of ISGs relative to untreated control cells (Fig 3A). Genes that were upregulated in both cGAMP-treated cells, and cells treated with the combination of VB-85680 and VACV-70 is described in Table 3. A full list of the top upregulated genes for each group are included in S1–S3 Tables.

**Table 3:** ISGs upregulated in both cGAMP-treated cells and cells treated with both VB-85680 and VACV-70. Upregulated ISGs and associated functions that were found to be in common between cGAMP-treated cells and cells treated with both VB-85680 and VACV-70.

Gene	Full gene name	Role in immune regulation
CMPK2	Cytidine/Uridine Monophosphate Kinase 2	nucleotide synthesis salvage; terminal differentiation of monocytic cells
DDX58	RIG1	type-I interferon response
HERC5	HECT And RLD Domain Containing E3 Ubiquitin Protein Ligase Family Member 5	response to virus
HERC6	HECT And RLD Domain Containing E3 Ubiquitin Protein Ligase Family Member 6	antigen processing and presentation
IFI27	Interferon Alpha Inducible Protein 27	cytokine signaling
IFI44	Interferon-Induced Protein 44	immune response.
IFI44L	Interferon-Induced Protein 44-Like	response to virus
IFI6	Interferon Alpha-Inducible Protein 6	response to virus
IFIT1	Interferon-Induced Protein With Tetratricopeptide Repeats 1	response to virus
IFIT2	Interferon-Induced Protein With Tetratricopeptide Repeats 2	cytokine signaling; interferon- mediated signaling
IFIT3	Interferon-Induced Protein With Tetratricopeptide Repeats 3	cytokine signaling; interferon- mediated signaling
IFIT5	Interferon-Induced Protein With Tetratricopeptide Repeats 5	cytokine signaling; interferon- mediated signaling
IFITM1	Interferon Induced Transmembrane Protein 1	response to virus
ISG15	ISG15 Ubiquitin Like Modifier	response to virus
ISG20	Interferon Stimulated Exonuclease Gene 20	response to virus
OAS2	2’-5’-Oligoadenylate Synthetase 2	response to virus
OAS3	2’-5’-Oligoadenylate Synthetase 3	response to virus
`OASL	2’-5’-Oligoadenylate Synthetase Like	response to virus
TRIM22	Tripartite Motif Containing 22	response to virus
USP18	Ubiquitin Specific Peptidase 18	downregulating interferon responses

Gene ontology (GO) analysis revealed that the majority of differentially-regulated genes identified in both cGAMP treated cells and cells treated with VB-85680 and VACV-70, are enriched for terms related to IFN response including innate immune response, Type-I interferon responses, defense to virus, and responses to viruses (Fig 3B). Although upregulated genes in VB-85680-treated cells did not reach predetermined criteria for significance, the top genes were also ISG-related genes (S3 Table) and GO analysis of the VB-85680-treated cells is also consistent with IFN response (S2 Fig).

**Fig 4:**
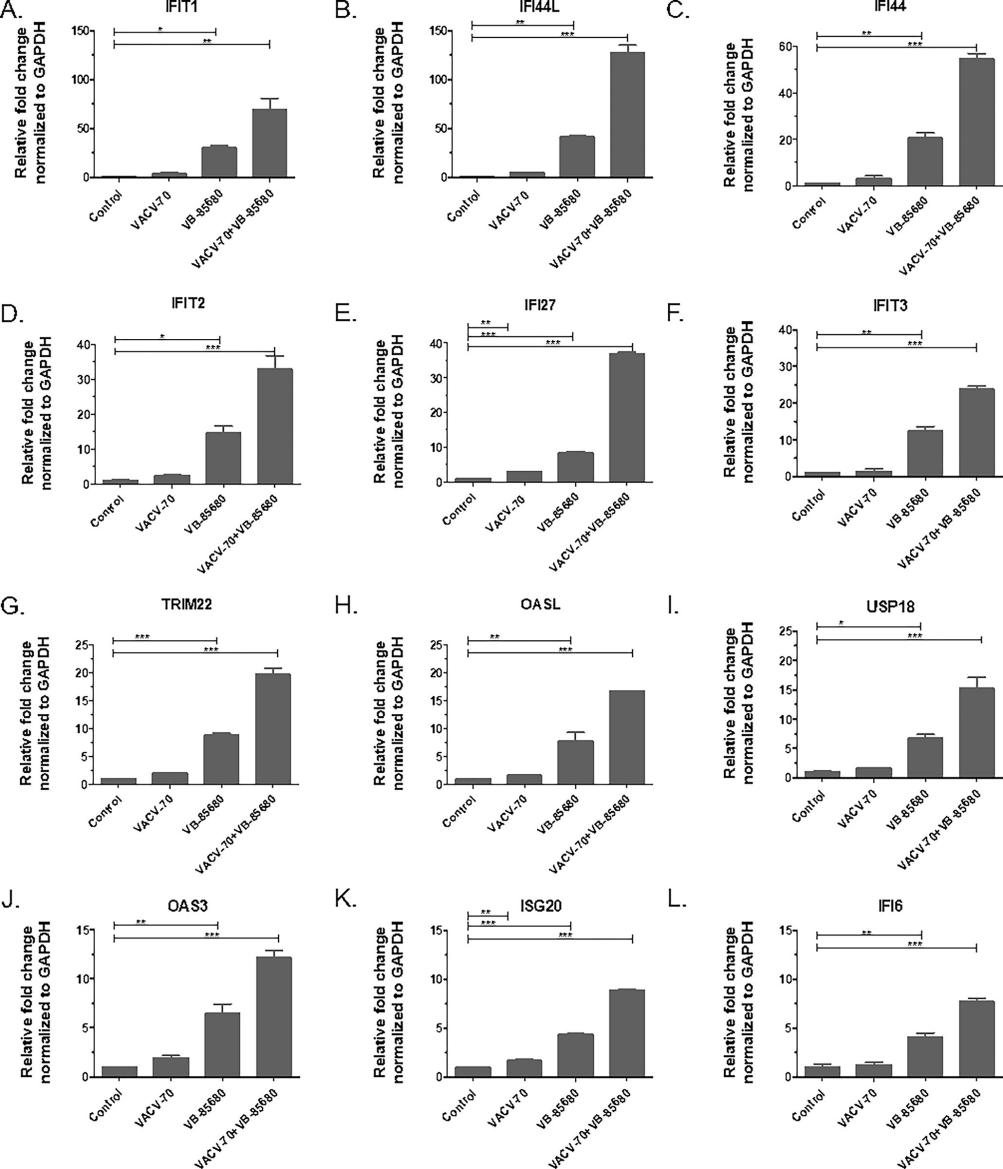
RT-qPCR confirmation of selective upregulated ISGs identified from RNA sequencing. **A-L)** Induction of key ISGs identified by RNAseq was confirmed using RT-qPCR. THP1-Dual™ cells were treated with either 1200 ng/mL VACV-70, 10 μM VB-85680, or a combination of the two. Expression was normalized to GAPDH and fold-change was calculated relative to the control sample. One-way ANOVA + Tukey’s Multiple Comparison Test was used to determine statistical significance. For significance, * = P ≤ 0.05, ** = P ≤ 0.01 and *** = P ≤ 0.001. Error bars in all experiments represent +/- SD.

To confirm RNAseq results, we used RT-qPCR to evaluate mRNA expression levels of 12 ISGs upregulated in both the VB-85680 and VACV-70 combination treatment, and the cGAMP treatment groups (Fig 4A–4L). As expected, the increase in mRNA level for each gene was greater in cells treated with both VB-85680 and VACV-70, compared to cells treated with VACV-70 or compound alone. In cells treated with VB-85680 alone, expression was generally higher than in cells treated only with VACV-70. This likely reflects cellular response to increased levels of cytosolic dsDNA and ssDNA in THP1-Dual™ cells following TREX1 inhibition.

### IFIT1 protein expression is upregulated in THP1-Dual™ cells treated with VB-85680 in combination with VACV-70 DNA

**Fig 5:**
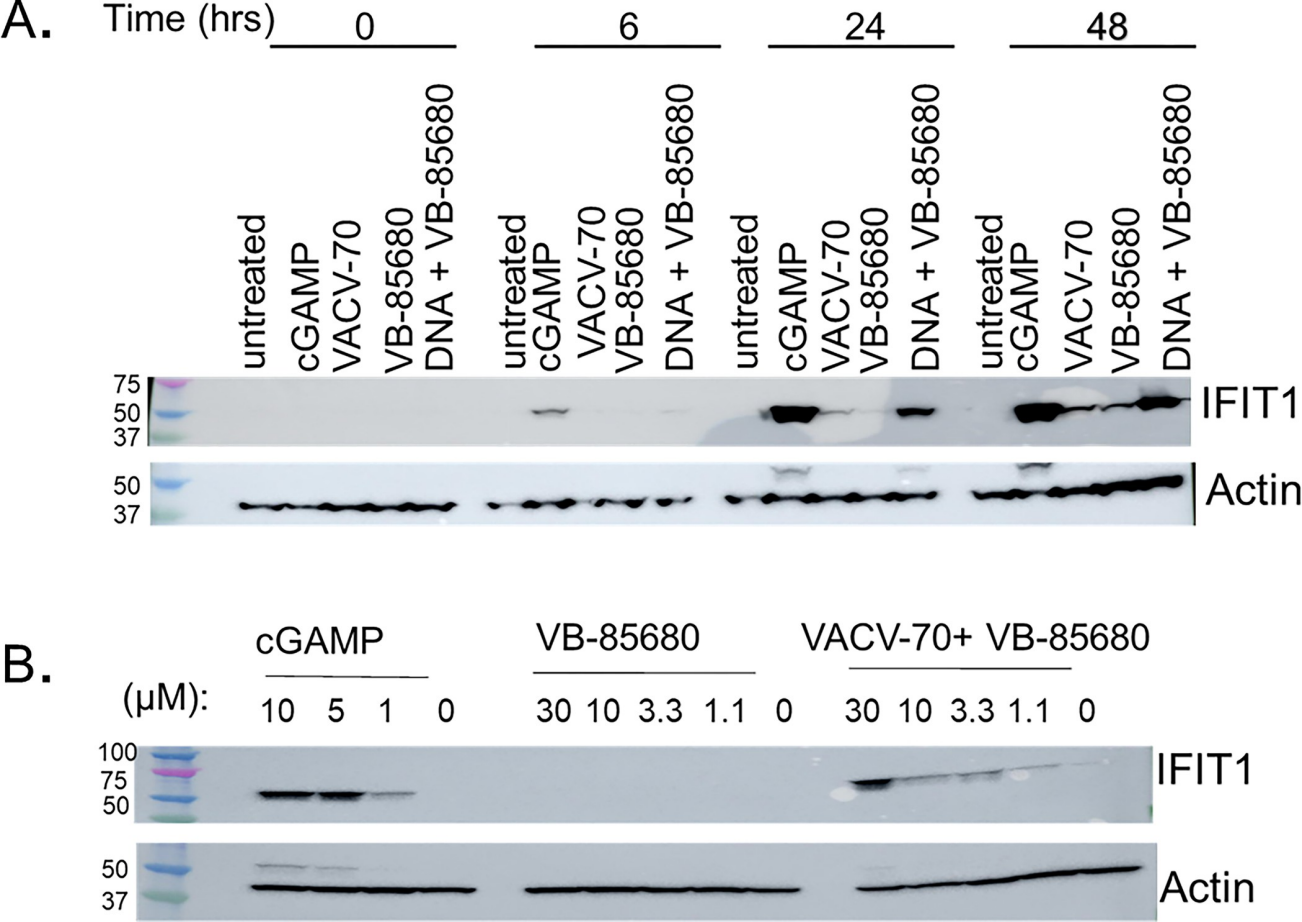
IFIT1 expression is upregulated in THP1-Dual™ treated by combination of VB-85680 and DNA. Expression of IFIT1 in THP1-Dual™ cells treated with DNA alone or in combination with VB-85680 was measured by Western blot. **A)** Time-course of IFIT1 expression in THP1-Dual™ cells treated with 1200 ng/mL VACV-70, 10 μM VB-85680, or both. cGAMP (10 μM)-treated THP1-Dual™ cells were used as a positive control. The combination of VB-85680 and VACV-70 increased the expression of IFIT1. **B)** THP1-Dual™ cells were treated with varying concentrations of VB-85680 in the presence or absence of VACV-70 for 24 hours. Cells were collected and subjected to Western blot analysis of IFIT1 expression. IFIT1 expression was only seen in the presence of TREX1 inhibitor and DNA stimulus. Actin was used as a loading control.

IFITs, including IFIT1, are amongst the most abundantly expressed ISG proteins. We wished to determine whether the upregulation of ISG mRNA was accompanied by a corresponding elevation in protein production. To this end, IFIT1 protein expression was evaluated by Western blot. Timing of IFIT1 protein production was first probed in THP1-Dual™ cells treated with either 10 μM cGAMP, 10 μM VB-85680, 1200 ng/mL VACV-70, or a combination of VB-85680 and VACV-70 (Fig 5A). Small amounts of IFIT1 were evident at 24 and 48 hours with either agent alone, or IFIT1 levels were markedly increased over either VACV-70 or VB-85680 alone, at both timepoints.

IFIT1 responsiveness to VB-85680 treatment in THP1-Dual™ cells was also observed to be dose-dependent. THP1-Dual™ cells were treated with varying doses of VB-85680 ranging from 1.1 to 30 μM with and without transfection of 1200 ng/mL VACV-70 for 24 hours. In the VB-85680-only cells, IFIT1 protein was undetectable by western blot (Fig 5B). In contrast, cells transfected with VACV-70 showed a dose-responsive increase in IFIT1 protein upon treatment with VB-85680.

## Discussion

Inhibitors targeting TREX1 are expected to reverse the dampening effect of TREX1 on the cGAS-STING pathway and reestablish IFN production and T cell priming. We have identified and characterized a novel small-molecule inhibitor of TREX1, VB-85680. VB-85680 inhibited the biochemical activity of full-length mouse TREX1, but had no effect on the enzymatically inert D18N TREX1 mutant. VB-85680 was also confirmed to inhibit endogenous mouse and human TREX1 activity in whole-cell lysates. VB-85680 treatment of living cells transfected with either G3-YSD or VACV-70 DNA changed ISG reporter activity or mRNA expression in human THP1 cells in a manner consistent with the induction of a Type-I IFN response via the cGAS-STING pathway, suggesting that VB-85680 may be capable of inhibiting intracellular TREX. In the course of characterizing VB-85680, we developed a cellular assay that may be useful in drug discovery efforts in the field.

Several studies highlight that modulation of TREX1 expression impacts immune response. Single doses of radiation greater than 12–18 Gy can induce TREX1, leading to reduced immunogenicity and IFN secretion [23]. However, repeated dosing below the threshold for increasing TREX1 expression, results in the stimulation of IFN production. In a separate study, direct microRNA targeting of TREX1 restored the immunogenicity of tumor cells and increased the secretion of proinflammatory factors into the tumor microenviroment [24]. Prati *et al* demonstrated that silencing TREX1 in HPV-positive cervical cancers inhibited tumor growth through the induction of p53 and subG1 accumulation [20]. Finally, loss of TREX1 in THP1 tumor cells increased tumor cell-derived Type-I IFN mRNA induction and the activation of DCs in response to the DNA damaging agent PBD SG-3199 [35]. In TREX1-deficient cells, cytosolic ssDNA resulting from aberrant DNA replication by-products can stimulate an immune response [36]. High levels of ssDNA in cancer cells have also been demonstrated to enhance the efficiency of immune checkpoint blockade (ICB) [37, 38].

Interestingly, a recent study published by Zhang *et al* suggests a mechanism by which a TREX1 inhibitor may function independently of the cGAS-STING pathway [39]. In this study the authors genetically silenced TREX1 in triple negative breast cancer cells to increase levels of ssDNA and then evaluated promotion of tumor immunogenicity in response to ssDNA accumulation. Suppression of TREX1 resulted in an ssDNA-generated interferon response that was independent of STING. In this context, DDX3X was identified as the ssDNA sensor responsible for mediating downstream effects. It was further shown that TREX1 depletion in murine models of breast cancer increased immune cell recruitment and response to ICB. This suggests that generation of cytosolic DNA through pharmacological TREX1 inhibition, could be used in conjunction with ICB therapy to enhance overall tumor immunogenicity.

In the present study, a small-molecule inhibitor of TREX1 exonuclease activity was capable of activating a cellular interferon response, as demonstrated by both an increase in mRNA ISG signature expression and IFIT1 protein expression in THP1- Dual cells. Simpson *et al* [40] demonstrated that TREX1 D18N catalytic deficiency caused diminished IFN-I signaling and autoimmunity in mice. Using RNAseq analysis they assessed differences in gene expression in splenic cells isolated from TREX1 WT mice and TREX1 D18N catalytically deficient mice. The authors also found an increase of ISG related genes in TREX1 D18N mice when compared with wild type; the genes they found to be elevated overlapped with our RNA-seq data. Gene ontology analysis from that study demonstrated significant enrichment in signaling pathways associated with immune responses and response to viral infection, again overlapping our data. Notably, gene ontology analysis of the RNAseq experiment from the present study was also enriched for the same pathways in cells treated with compound alone or in combination with VACV-70. Thus, our approach of pharmacological TREX1 inhibition mimicked the functional disruption of TREX1 through genetic approaches, shown by Simpson *et al* [40].

A novel small-molecule TREX1 inhibitor may be of use in treatment of malignant disease, particularly if used in conjunction with chemotherapeutic or radiotherapeutic modalities that generate cytoplasmic DNA which would otherwise be cleared by TREX1. VB-85680 elicits strong Type-I IFN signaling and ISG signature and so might reasonably be expected to enhance anti-tumor innate and adaptive immune responses. Strong anti-tumor effects may also be possible with TREX1 inhibitor monotherapy in tumors with intrinsic chromosomal instability [41].

In conclusion, we identified a potent *in vitro* inhibitor of TREX1 exonuclease activity, VB-85680. We further defined cellular assay conditions which show that VB-85680 can activate cGAS-STING pathway signaling. The compound also elicits in THP-1 cells a strong Type-I IFN signaling and ISG signature known to have an important role in both innate and anti-tumor immunity. TREX1 inhibitors are expected to enhance tumor immunogenicity under a variety of therapeutic approaches, and may well function to overcome immunosuppressive effects of otherwise efficacious radiation therapy. Further investigation is needed to determine whether the methods described herein will prove useful for evaluation of functional activity of VB-85680 analogs and aid in the identification of a suitable candidate to move forward to *in vivo* proof-of-concept studies.

## Supporting information

S1 Fig Dose response activity of TREX1 VB-86087 and G150 in THP1-Dual™. (PDF)

S2 Fig GO analysis of THP1-Dual™ cells treated with VB-85680 relative to untreated control. (PDF)

S1 Table Top upregulated genes in cGAMP-Treated THP1-Dual™ cells relative to untreated control. (PDF)

S2 Table Top upregulated genes in THP1-Dual™ cells treated with VB-85680 and VACV-70. (PDF)

S3 Table Top 28 upregulated genes in untreated vs VB-85680 Treated THP1- Dual™ cells and GO analysis. (PDF)
