# Supplementary material for: A novel TREX1 inhibitor, VB-85680, upregulates cellular interferon responses
Source: PLoS One. 2024 Aug 23;19(8):e0305962. doi: 10.1371/journal.pone.0305962 (PMC11343403; doi:10.1371/journal.pone.0305962)
Supplement: S1 Fig — (PDF) [file pone.0305962.s001.pdf]

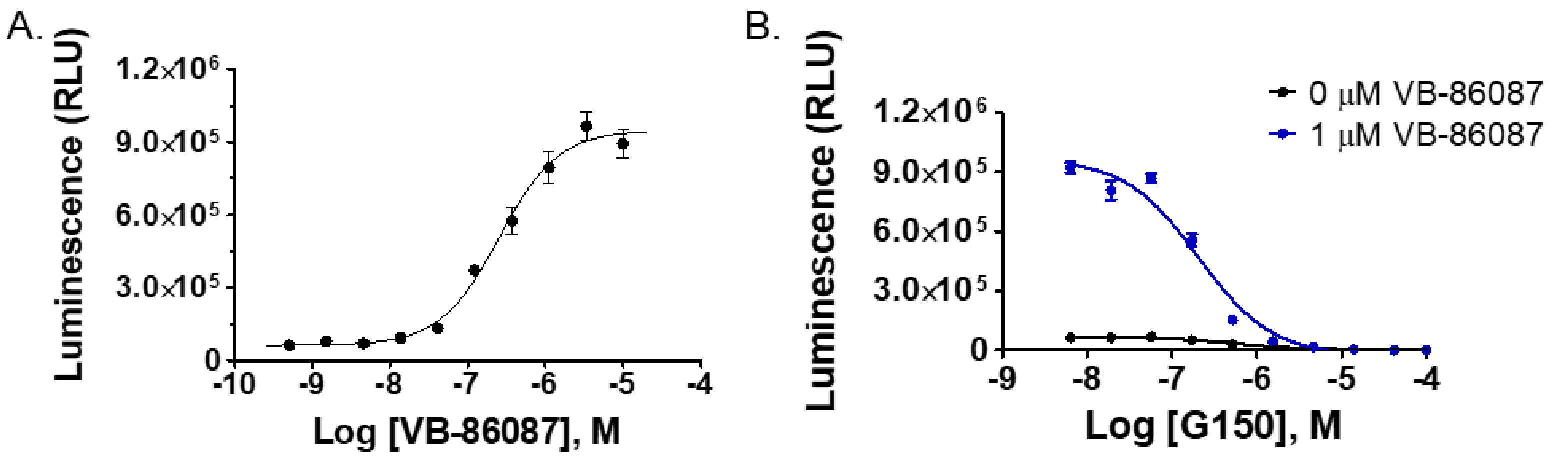

Supplemental Figure 1: Dose response activity of TREX1 VB-86087 and G150 in THP1-Dual™ cells. **A)** THP1-Dual™ cells were cultured for 3 days under low serum conditions. The cells were then batch-transfected with 10 ng/mL G3-YSD and treated with decreasing doses of VB-86087, starting at 30 µM. The EC_{50} of VB-86087 was determined to be 0.25 µM. **B)** Titration of cGAS inhibitor G150 in THP1-Dual™ cells. THP1-Dual™ cells were cultured as described above were treated with decreasing doses of G150, starting at 100 µM, and in the presence of 1 µM of the TREX1 inhibitor VB-86087. Error bars for all experiments represent +/- SD.
