# Supplementary material for: A novel TREX1 inhibitor, VB-85680, upregulates cellular interferon responses
Source: PLoS One. 2024 Aug 23;19(8):e0305962. doi: 10.1371/journal.pone.0305962 (PMC11343403; doi:10.1371/journal.pone.0305962)
Supplement: S2 Fig — (PDF) [file pone.0305962.s002.pdf]

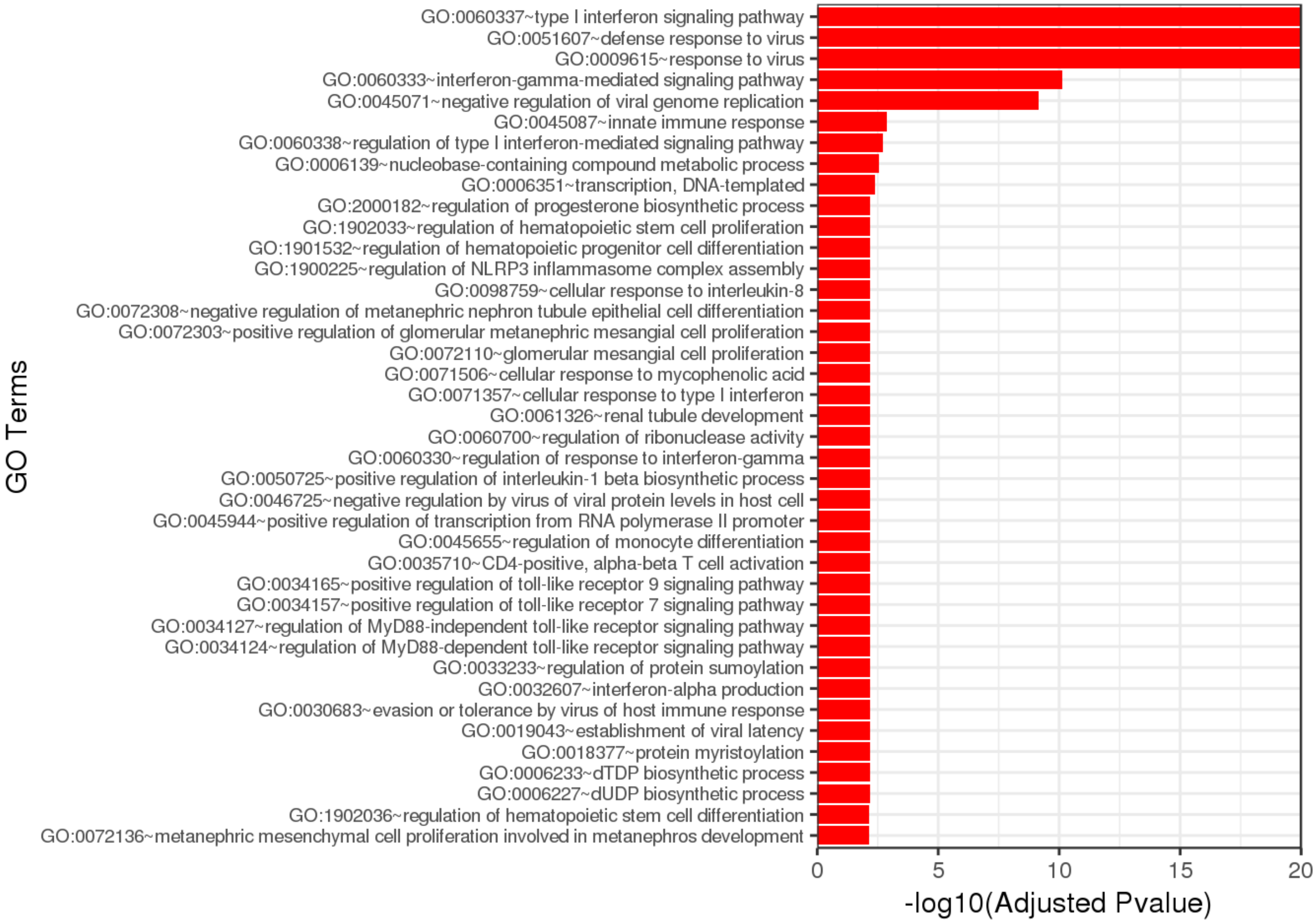

Supplemental Figure 2: GO analysis of THP1-Dual™ cells treated with VB-85680 alone shows an enrichment of immune related pathways similar to cGAMP treated cells. Gene ontology (GO) analysis of THP1-Dual™ cells treated with VB-85680 alone
