# Supplementary material for: A novel TREX1 inhibitor, VB-85680, upregulates cellular interferon responses
Source: PLoS One. 2024 Aug 23;19(8):e0305962. doi: 10.1371/journal.pone.0305962 (PMC11343403; doi:10.1371/journal.pone.0305962)
Supplement: S1 Table — (PDF) [file pone.0305962.s003.pdf]

Top Upregulated genes-Untreated vs cGAMP				
**GeneID**	**GeneName**	**log2FoldChange**	**pvalue**	**padj**
ENSG00000108700	CCL8	5.2	3.3E-06	5.1E-05
ENSG00000182393	IFNL1	4.7	9.5E-07	1.7E-05
ENSG00000225964	NRIR	4.3	1.2E-18	5.9E-17
ENSG00000136960	ENPP2	4.2	3.6E-08	7.9E-07
ENSG00000133328	HRASLS2	4.2	4.5E-08	9.7E-07
ENSG00000171855	IFNB1	4.1	3.4E-15	1.4E-13
ENSG00000057704	TMCC3	4.1	3.6E-15	1.5E-13
ENSG00000137959	IFI44L	4.0	4.9E-29	3.9E-27
ENSG00000169245	CXCL10	4.0	2.1E-36	2.2E-34
ENSG00000169248	CXCL11	4.0	2.0E-53	3.4E-51
ENSG00000154451	GBP5	3.9	3.3E-29	2.7E-27
ENSG00000172183	ISG20	3.9	2.5E-46	3.6E-44
ENSG00000185745	IFIT1	3.9	2.3E-44	3.0E-42
ENSG00000163568	AIM2	3.9	7.0E-34	6.6E-32
ENSG00000117228	GBP1	3.9	1.6E-29	1.3E-27
ENSG00000225492	GBP1P1	3.8	9.2E-22	5.4E-20
ENSG00000119917	IFIT3	3.8	5.6E-55	1.0E-52
ENSG00000119922	IFIT2	3.7	1.1E-39	1.2E-37
ENSG00000137965	IFI44	3.7	3.2E-34	3.1E-32
ENSG00000135114	OASL	3.7	6.2E-31	5.3E-29
ENSG00000165949	IFI27	3.7	3.7E-42	4.4E-40
ENSG00000244694	PTCHD4	3.7	1.5E-07	3.0E-06
ENSG00000204616	TRIM31	3.6	1.1E-06	1.9E-05
ENSG00000147050	KDM6A	3.6	1.3E-06	2.2E-05
ENSG00000111335	OAS2	3.6	6.2E-35	6.2E-33
ENSG00000111331	OAS3	3.6	9.0E-31	7.6E-29
ENSG00000079385	CEACAM1	3.6	3.2E-08	7.2E-07
ENSG00000138646	HERC5	3.5	1.5E-33	1.4E-31
ENSG00000184979	USP18	3.5	9.9E-36	1.0E-33
ENSG00000090530	P3H2	3.5	2.5E-24	1.6E-22
ENSG00000132274	TRIM22	3.5	3.4E-59	6.6E-57
ENSG00000135333	EPHA7	3.5	3.6E-35	3.7E-33
ENSG00000183813	CCR4	3.5	3.8E-14	1.5E-12
ENSG00000133106	EPSTI1	3.5	2.6E-66	5.9E-64
ENSG00000271474	AC106881.1	3.5	4.6E-06	7.1E-05
ENSG00000187608	ISG15	3.4	0.0E+00	0.0E+00
ENSG00000134326	CMPK2	3.4	0.0E+00	0.0E+00
ENSG00000089692	LAG3	3.4	2.1E-14	8.3E-13
ENSG00000185885	IFITM1	3.3	1.6E-69	3.8E-67
ENSG00000152778	IFIT5	3.3	5.0E-38	5.5E-36
ENSG00000181634	TNFSF15	3.3	3.7E-28	2.9E-26
ENSG00000115155	OTOF	3.3	2.1E-07	4.1E-06
ENSG00000126709	IFI6	3.3	7.7E-295	2.2E-291
ENSG00000108387	SEPT4	3.2	2.1E-21	1.2E-19
ENSG00000225889	AC012368.1	3.3	7.2E-10	1.9E-08
ENSG00000145147	SLIT2	3.2	1.7E-06	2.8E-05
ENSG00000107201	DDX58	3.2	6.3E-43	7.7E-41
ENSG00000138642	HERC6	3.2	1.6E-191	2.6E-188
ENSG00000010030	ETV7	3.2	1.3E-58	2.6E-56
ENSG00000225886	AL445490.1	3.2	1.0E-06	1.7E-05

Supplemental Table 1: Top upregulated genes in cGAMP-Treated THP1-Dual™ cells relative to untreated control. The top upregulated genes in cGAMP treated THP1- Dual™ cells compared to untreated cells sorted by their log2 fold change.
