# Supplementary material for: A novel TREX1 inhibitor, VB-85680, upregulates cellular interferon responses
Source: PLoS One. 2024 Aug 23;19(8):e0305962. doi: 10.1371/journal.pone.0305962 (PMC11343403; doi:10.1371/journal.pone.0305962)
Supplement: S2 Table — (PDF) [file pone.0305962.s004.pdf]

Top Upregulated geness- Untreated vs VB-85680 + VACV-70				
**GeneID**	**GeneName**	**log2FoldChange**	**pvalue**	**padj**
ENSG00000137959	IFI44L	3.9	7.8E-28	1.3E-25
ENSG00000111331	OAS3	3.3	8.0E-26	1.1E-23
ENSG00000111335	OAS2	3.3	2.8E-30	5.0E-28
ENSG00000137965	IFI44	3.2	2.5E-25	3.4E-23
ENSG00000132274	TRIM22	3.2	3.1E-142	1.2E-138
ENSG00000185745	IFIT1	3.1	8.8E-29	1.5E-26
ENSG00000133106	EPSTI1	3.1	3.3E-139	9.6E-136
ENSG00000184979	USP18	3.0	2.2E-26	3.3E-24
ENSG00000134326	CMPK2	2.9	4.3E-152	2.5E-148
ENSG00000187608	ISG15	2.9	6.7E-119	8.6E-116
ENSG00000138642	HERC6	2.8	2.4E-95	1.5E-92
ENSG00000088827	SIGLEC1	2.8	5.3E-28	8.6E-26
ENSG00000135333	EPHA7	2.8	1.3E-17	1.3E-15
ENSG00000177409	SAMD9L	2.8	4.9E-28	8.2E-26
ENSG00000185885	IFITM1	2.8	1.4E-120	2.7E-117
ENSG00000126709	IFI6	2.8	7.4E-124	1.7E-120
ENSG00000165949	IFI27	2.8	3.0E-21	3.4E-19
ENSG00000138496	PARP9	2.7	2.9E-120	4.8E-117
ENSG00000089127	OAS1	2.7	4.1E-100	3.4E-97
ENSG00000185507	IRF7	2.7	2.1E-153	2.4E-149
ENSG00000119917	IFIT3	2.6	8.2E-95	5.0E-92
ENSG00000152778	IFIT5	2.6	1.1E-73	4.8E-71
ENSG00000225963	AC009950.1	2.6	1.3E-21	1.5E-19
ENSG00000130589	HELZ2	2.6	3.2E-19	3.4E-17
ENSG00000167601	AXL	2.5	3.8E-16	3.4E-14
ENSG00000137628	DDX60	2.6	9.0E-17	8.5E-15
ENSG00000173193	PARP14	2.5	7.7E-94	4.4E-91
ENSG00000108771	DHX58	2.5	7.5E-91	3.9E-88
ENSG00000134321	RSAD2	2.5	1.5E-77	6.7E-75
ENSG00000135114	OASL	2.5	3.8E-16	3.4E-14
ENSG00000172183	ISG20	2.5	4.6E-50	1.2E-47
ENSG00000115415	STAT1	2.5	1.7E-104	1.7E-101
ENSG00000138646	HERC5	2.4	1.1E-63	3.7E-61
ENSG00000163666	HESX1	2.4	4.3E-36	8.3E-34
ENSG00000107201	DDX58	2.4	8.6E-76	3.8E-73
ENSG00000283648	AC006974.2	2.4	7.6E-17	7.3E-15
ENSG00000132530	XAF1	2.4	2.2E-49	5.6E-47
ENSG00000156587	UBE2L6	2.4	1.1E-119	1.6E-116
ENSG00000135899	SP110	2.4	8.6E-96	6.2E-93
ENSG00000183486	MX2	2.4	8.7E-114	1.0E-110
ENSG00000055332	EIF2AK2	2.4	9.0E-99	7.0E-96
ENSG00000119922	IFIT2	2.3	2.3E-17	2.3E-15
ENSG00000137198	GMPR	2.3	3.1E-30	5.5E-28
ENSG00000010030	ETV7	2.3	3.8E-23	4.8E-21
ENSG00000173821	RNF213	2.3	2.0E-95	1.4E-92
ENSG00000163565	IFI16	2.3	2.0E-89	9.9E-87
ENSG00000115267	IFIH1	2.3	2.3E-17	2.3E-15
ENSG00000162614	NEXN	2.3	6.6E-71	2.6E-68
ENSG00000205413	SAMD9	2.3	3.3E-67	1.2E-64
ENSG00000162654	GBP4	2.2	9.5E-43	2.0E-40

Supplemental Table 2: Top Upregulated genes in THP1-Dual™ cells treated with VB-85680 and VACV-70. The top genes upregulated in THP1-Dual™ cells treated with VB-85680 and VACV-70 relative to untreated cells sorted by their log2 fold change.
