# Supplementary material for: A novel TREX1 inhibitor, VB-85680, upregulates cellular interferon responses
Source: PLoS One. 2024 Aug 23;19(8):e0305962. doi: 10.1371/journal.pone.0305962 (PMC11343403; doi:10.1371/journal.pone.0305962)
Supplement: S3 Table — (PDF) [file pone.0305962.s005.pdf]

Top Upregulated genes- Untreated vs VB-85680				
**GeneID**	**GeneName**	**log2FoldChange**	**pvalue**	**padj**
ENSG00000111335	OAS2	1.7	1.6E-09	3.1E-07
ENSG00000177409	SAMD9L	1.6	3.9E-11	9.3E-09
ENSG00000138496	PARP9	1.5	1.9E-47	7.7E-44
ENSG00000185507	IRF7	1.4	1.1E-57	9.4E-54
ENSG00000152778	IFIT5	1.4	1.5E-21	8.2E-19
ENSG00000130589	HELZ2	1.4	7.8E-07	1.1E-04
ENSG00000055332	EIF2AK2	1.4	4.1E-47	9.2E-44
ENSG00000134326	CMPK2	1.4	7.4E-46	1.3E-42
ENSG00000089127	OAS1	1.4	8.4E-10	1.7E-07
ENSG00000137959	IFI44L	1.4	1.3E-04	9.3E-03
ENSG00000115415	STAT1	1.3	2.6E-47	7.7E-44
ENSG00000088827	SIGLEC1	1.3	2.1E-07	3.3E-05
ENSG00000135899	SP110	1.3	8.1E-34	1.0E-30
ENSG00000185745	IFIT1	1.3	1.9E-06	2.5E-04
ENSG00000173193	PARP14	1.3	5.5E-12	1.4E-09
ENSG00000111331	OAS3	1.3	1.9E-05	1.8E-03
ENSG00000137628	DDX60	1.3	9.7E-06	1.1E-03
ENSG00000138642	HERC6	1.3	3.2E-20	1.6E-17
ENSG00000133106	EPSTI1	1.2	1.6E-24	1.3E-21
ENSG00000205413	SAMD9	1.2	7.6E-08	1.3E-05
ENSG00000137965	IFI44	1.1	1.3E-04	9.4E-03
ENSG00000134321	RSAD2	1.1	1.6E-16	6.2E-14
ENSG00000173821	RNF213	1.1	6.4E-27	6.3E-24
ENSG00000184979	USP18	1.1	1.1E-04	8.6E-03
ENSG00000119917	IFIT3	1.0	5.8E-06	7.1E-04
ENSG00000282851	BISPR	1.0	1.3E-18	5.5E-16
ENSG00000132274	TRIM22	1.0	2.5E-15	8.6E-13
ENSG00000120738	DDX58	1.0	4.5E-05	3.9E-03

Supplemental Table 3: Top 28 Upregulated genes in untreated vs VB-85680 Treated THP1-Dual™ cells and GO analysis. The top upregulated expressed genes in VB-85680 treated THP1- Dual™ cells compared to untreated cells, sorted by their log2 fold-change.
